# Quality assurance for the EORTC 22071–26071 study: dummy run prospective analysis

**DOI:** 10.1186/s13014-014-0248-9

**Published:** 2014-11-26

**Authors:** Alysa Fairchild, Johannes A Langendijk, Sandra Nuyts, Christopher Scrase, Milan Tomsej, Danny Schuring, Akos Gulyban, Sunita Ghosh, Damien C Weber, Wilfried Budach

**Affiliations:** Department of Radiation Oncology, Cross Cancer Institute, 11560 University Avenue, T6G 1Z2 Edmonton, AB Canada; Department of Radiation Oncology, University of Groningen, University Medical Centre Groningen, Groningen, The Netherlands; Department of Radiation Oncology, University Hospital Leuven, KU Leuven, Leuven, Belgium; Department of Radiation Oncology, Ipswich Hospital, Ipswich, UK; Department of Radiotherapy, CHU Charleroi, Charleroi, Belgium; EORTC Quality Assurance in Radiotherapy Team, Brussels, Belgium; Department of Radiotherapy, Catharina Hospital, Eindhoven, The Netherlands; Department of Radiation Oncology, University Hospital of Liege, Liege, Belgium; Department of Experimental Oncology, Cross Cancer Institute, Edmonton, Canada; Centre for Proton Therapy, Paul Scherrer Institute, Villigen, Switzerland; Department of Radiation Oncology, Heinrich Heine University, Dusseldorf, Germany

**Keywords:** Head and neck, IMRT, Dummy run, EORTC ROG

## Abstract

**Purpose:**

The phase III 22071–26071 trial was designed to evaluate the addition of panitumumab to adjuvant chemotherapy plus intensity modulated radiotherapy (IMRT) in locally advanced resected squamous cell head and neck cancer. We report the results of the dummy run (DR) performed to detect deviations from protocol guidelines.

**Methods and Materials:**

DR datasets consisting of target volumes, organs at risk (OAR) and treatment plans were digitally uploaded, then compared with reference contours and protocol guidelines by six central reviewers. Summary statistics and analyses of potential correlations between delineations and plan characteristics were performed.

**Results:**

Of 23 datasets, 20 (87.0%) GTVs were evaluated as acceptable/borderline, along with 13 (56.5%) CTVs and 10 (43.5%) PTVs. All PTV dose requirements were met by 73.9% of cases. Dose constraints were met for 65.2-100% of mandatory OARs. Statistically significant correlations were observed between the subjective acceptability of contours and the ability to meet dose constraints for all OARs (p ≤ 0.01) except for the parotids and spinal cord. Ipsilateral parotid doses correlated significantly with CTV and PTV volumes (p ≤ 0.05).

**Conclusions:**

The observed wide variations in treatment planning, despite strict guidelines, confirms the complexity of development and quality assurance of IMRT-based multicentre studies for head and neck cancer.

**Electronic supplementary material:**

The online version of this article (doi:10.1186/s13014-014-0248-9) contains supplementary material, which is available to authorized users.

## Background

The management of locally advanced head and neck squamous cell carcinoma (HNSCC) involves increasingly complex combined modality approaches. After primary surgery, conventionally-fractionated adjuvant radiotherapy (RT), commonly delivered to a total dose of 64-66 Gy, reduces the locoregional recurrence rate by at least half, which has translated into a survival benefit [1-8]. The addition of concurrent cisplatin has been investigated in two major randomized trials with significant improvements in progression-free [6,7] and overall survival (OS) [6] overall, and for the subgroup of patients with extra-capsular extension (ECE) and/or surgical margins <5 mm [9]. Additionallly, blockade of the epidermal growth factor receptor (EGFR) with cetuximab reduces the likelihood of disease progression and increases 3-year OS by 10% without enhancing typical RT side effects [10]. The open-label, multicentre randomized phase III EORTC 22071 trial was designed to determine whether adding the EGFR inhibitor panitumumab to adjuvant chemoradiotherapy (CRT) concurrently would significantly prolong disease-free survival (DFS) in macroscopically completely resected HNSCC (ClinicalTrials.gov NCT01142414). Eligible patients had surgically resected non-metastatic squamous cell carcinoma of the hypopharynx, oropharynx, larynx or oral cavity, stage pT1-2 node positive, or any pT3-4 (UICC 6^th^ Edition) at high risk of locoregional recurrence based on one or more of the following: R0 resection with surgical margins <5 mm, R1 resection (margin <1 mm) or ECE.

Variations in compliance with protocol RT delivery in multicentre studies decrease tumour control, increase RT toxicity and may negatively impact survival [11-19]. An extensive quality assurance (QA) program increases inter-institutional consistency and familiarizes participating sites with EORTC procedures [16]. Centres undergoing pre-trial accreditation are better prepared to comply with protocol requirements [17], since QA ensures participating institutions can adhere to protocol instructions, including adequate contouring of volumes [18]. In a secondary analysis of available Radiation Oncology Group dummy run (DR) cases over two decades, institutions which previously completed a DR were significantly more likely to be successful at other trials’ QA [19].

Planned RT QA procedures for EORTC 22071 included completion of a trial-specific DR as well as a complex dosimetry check (phantom irradiation). However, in spring 2011, the RTOG reported that adding concurrent cetuximab to CRT did not benefit patients with locally advanced unresected HNSCC [20]. Therefore, testing the same concept in the adjuvant setting with a different investigational agent was not considered likely to be beneficial. As a consequence, the trial was suspended in July 2011 prior to accrual of its first patient. Despite closure of the trial, we analyzed completed DR datasets to assess compliance with protocol guidelines.

## Methods

### DR procedure

**Figure 1:**
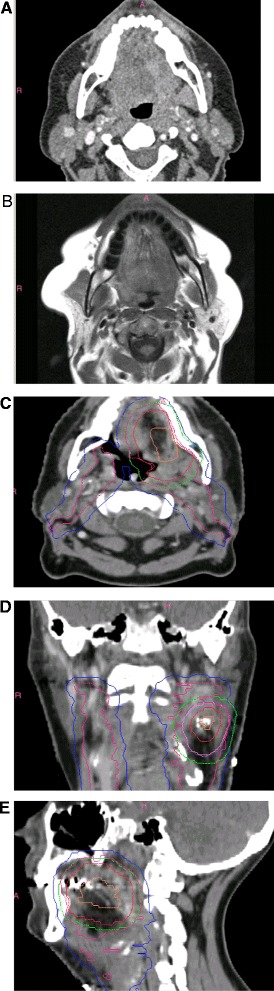
**Dummy run case imaging.** Representative preoperative **(A)** computed tomography and **(B)** axial T2-weighted magnetic resonance image provided. (**C)** Axial, **(D)** coronal and **(E)** sagittal display of master contours. Orange: GTV-pt. Magenta: CTV52.8 Gy. Pink: CTV59.4 Gy. Red: CTV66 Gy. Blue: PTV52.8 Gy-Exact*. Green: PTV2Exact*. Purple: PTV3Exact*. *PTVExacts denote PTVs collapsed inside the skin by 5 mm.

The DR case history (Online Additional file 1) reflected a 51 year old female with a pT2N1M0 left lateral tongue HNSCC post-R1 resection (Figure 1). CT simulation imaging and preoperative CT and MRI scans were downloaded from the EORTC by participating institutions and loaded into the local treatment planning systems (TPS). Target volumes and organs at risk (OAR) were to be defined and a protocol-compliant treatment plan generated. Subsequently, the planning CT in DICOM format, plus the structure set, 3D dose matrix and RT plan (DICOM-RT format) were uploaded to the EORTC Quality Assurance in Radiotherapy office via a web-based tool. After evaluation for data integrity, datasets were transmitted to the Image-Guided Therapy Quality Assurance Centre (http://atc.wustl.edu) for review by the trial QA team in relation to the master contours. Master contours had been constructed independently by four expert head and neck radiation oncologists according to protocol guidelines (WB, JL, SN, CS), after which two meetings were held to reach consensus.

### Radiotherapy

The protocol recommended that the diagnostic CT be co-registered with the postoperative planning CT to facilitate definition of the preoperative extent of primary tumour (“GTV-pt”). A low risk volume (CTV52.8 Gy), and areas at intermediate (CTV59.4 Gy) and high risk (CTV66 Gy) of harbouring microscopic disease were to be defined (Table 1). Volume selection guidelines were outlined in the protocol, based on Gregoire et al. [21]. Although sites were given the option to encompass the entire LN level containing the LN with ECE for trial patients, for the DR patient, the preoperative extent of the LN with ECE was considered to be clearly identifiable. Therefore, only the reconstructed LN with ECE should have been included. For all CTVs, 3D margin expansion was to be anatomically adapted to avoid structures not at risk for microscopic disease (eg air cavity, bone). Three PTVs were to be generated using a recommended margin of 5 mm; 3 mm was allowed if using advanced position verification procedures. Required optimization structures called ‘PTV-Exacts’ were equivalent to the respective PTVs collapsed inside the external body contour by 5 mm to avoid extension into the build-up region. A simultaneous integrated boost (SIB) technique with 6-10MV photons was required for all patients receiving intensity modulated RT (IMRT). Prescription dose to the PTV1-Exact was 52.8 Gy (referred to throughout as “PTV52.8 Gy-Exact”), PTV2-Exact was 59.4 Gy (“PTV59.4 Gy-Exact”), and PTV3-Exact was 66.0 Gy (“PTV66 Gy-Exact”), each delivered in 33 fractions; at least 95% of the prescribed dose was to cover 95% of the PTV-Exacts.

**Table 1:** **Target volumes and normal tissues: objectives and constraints**

	**Protocol compliant**		**Minor deviation**	**Major deviation**
**Target structure**				
CTV52.8 Gy	• Bilateral neck irradiation to include levels IB, II, III and IV		• Level IA or V included	• Unilateral irradiation
				• Complete levels IB, II, III or IV not included
	• Preoperative extent of primary tumour +15 mm			• Preoperative extent of tumour or LN with ECE outside of CTV
	• Reconstructed LN with ECE +10 mm			
CTV59.4 Gy	• Preoperative extent of primary tumour +10 mm		--	• Preoperative extent of tumour or LN with ECE outside of CTV
	• Reconstructed LN with ECE +10 mm			
CTV66 Gy	• Preoperative extent of primary tumour +10 mm		--	• Preoperative extent of tumour or LN with ECE outside of CTV
	• Reconstructed LN with ECE +5 mm			
	**Volume objective**	**Dose**		
PTV52.8 Gy-Exact	D98%	≥47.52 Gy	46.57-47.52 Gy	<46.57 Gy
	D95%	≥50.16 Gy	49.16-50.16 Gy	<49.16 Gy
PTV59.4 Gy-Exact	D98%	≥53.46 Gy	52.93-53.46 Gy	<52.93 Gy
	D95%	≥56.43 Gy	55.30-56.43 Gy	<55.30 Gy
PTV66 Gy-Exact	D98%	≥59.4 Gy	58.21-59.40 Gy	<58.21 Gy
	D95%	≥62.7 Gy	61.45-62.70 Gy	<61.45 Gy
	D5%	≤72.6 Gy	--	>72.6 Gy
**Normal tissue**	**Dose constraint**			
Outside of PTV3*	Max dose 72.6 Gy to a volume >1.8 cc		>72.6 Gy to >1.8-2.0 cc	>72.6 Gy to >2.0 cc
Spinal cord*	D2%	<50 Gy	--	≥50 Gy
Brainstem*	D2%	<52 Gy	--	≥52 Gy
Parotid glands**				
• Either gland	Mean dose	<26 Gy	--	≥26 Gy
• Either gland	≥50%	<30 Gy	--	≥30 Gy
• Both glands combined	≥20 cc	<20 Gy	--	≥20 Gy
Submandibular gland	Mean dose	<52 Gy	--	≥52 Gy
Oral cavity	Max dose outside PTV 50 Gy		--	≥50 Gy
Larynx	Max dose outside PTV 50 Gy		--	≥50 Gy
Other^	As low as possible		--	--

*Mandatory dose limits. **Investigators must meet at least one of three constraints. ^Optic nerves, chiasm, lenses, mandible, globes, brachial plexus, cochleae etc. *Abbreviation*: *D2%* near max dose, *ECE* extracapsular extension, *LN* lymph node, *max* maximum.

### Organs at risk

Delineation of the brainstem, spinal cord (equal to the osseous borders of vertebral canal), and parotid glands was mandatory. All other OARs were considered optional. Formal planning volume at risk margins were not utilized.

### Central review

Four radiation oncologists and two medical physicists participated in the central review procedure. Reviewers evaluated volume selection and delineation in relation to master contours (Figure 1), as well as treatment planning parameters, OAR dosimetry, dose distributions and dose-volume histograms (DVH). Deviations were graded as acceptable, borderline, and unacceptable based on the defintions in Table 1 and taking into account all available information along with ICRU recommendations [22]. For example, the final grade of the CTVs considered the degree of compliance of the GTV. In case of discrepant judgments of reviewers, the grade was assigned either by WB or by JL. Based on all reviewer comments, an overall grade was given to each DR dataset by WB or JL.

### Statistical analysis

Descriptive statistics were compiled as proportions for categorical variables, and averages (standard deviations) for normally distributed continuous variables. Recalculated DVHs were used to assess dosimetric parameters. Fisher’s exact test evaluated the correlation between two categorical variables with cell count <5. The Spearman correlation coefficient explored correlations between characteristics of target volumes and OAR doses. The independent *t*-test compared OAR doses delivered to volumes which did versus did not meet protocol constraints. A p value of ≤0.05 was considered statistically significant. All analysis was conducted using IBM SPSS version 19. The mean virtual radius was obtained for all CTVs by calculating the radius of a sphere with the identical volume as the corresponding CTV.

## Results

Twenty-three DR cases were submitted from investigators in nine countries (10/2010-11/2011). All were IMRT plans using dynamic multileaf collimation (34.8%), helical tomotherapy (21.7%), static multileaf collimation (17.5%), volumetric modulated arc therapy (13.0%) or technique not specified (13.0%). TPS included Eclipse (52.5%), Tomotherapy HiART (17.4%), Pinnacle (8.7%), Xio (8.7%), Monaco (4.3%), and not specified (8.8%). Eleven (47.8%) sites successfully completed the phantom irradiation for IMRT credentialing prior to submission of the DR case. A median of 28 contours were submitted per DR dataset (range 15–47) including optional planning optimization structures.

### Volume delineation

**Figure 2:**
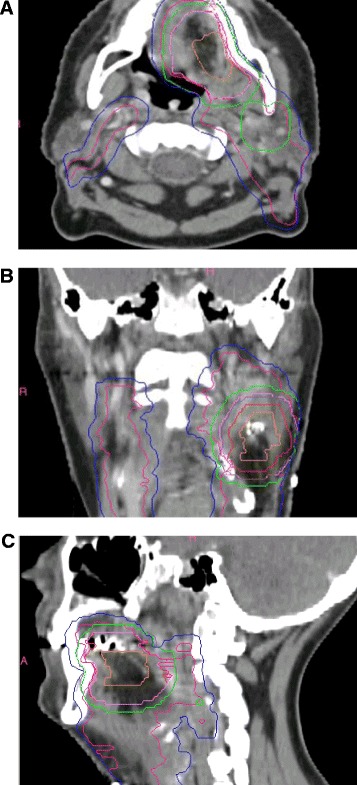
**Example of a submitted dataset with contours graded overall as minor deviation, (A) axial, (B) coronal and (C) sagittal views.** Superior border of GTV-pt (orange) and CTVs insufficiently high. CTV52.8 Gy (magenta) should not extend outside platysma or into submental, pretracheal or sternocleidomastoid regions. PTVs should be carved out of normal structures oral cavity and larynx. Orange: GTV-pt. Magenta: CTV52.8 Gy. Pink: CTV59.4 Gy. Red: CTV66 Gy. Blue: PTV52.8 Gy-Exact*. Green: PTV2Exact*. Purple: PTV3Exact*. *PTVExacts denote PTVs collapsed inside the skin by 5 mm.

**Figure 3:**
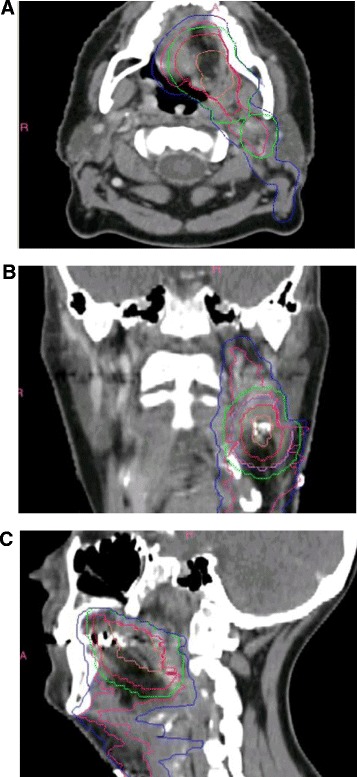
**Example of a submitted dataset with contours graded overall as major deviation, (A) axial, (B) coronal and (C) sagittal views.** GTV-pt not included in its entirety in all CTVs. Contralateral level IB-IV not included in CTV52.8 Gy, level V not adequately included, and level IA included erroneously. For normal tissue contours, medulla (instead of entire brainstem) and spinal cord (instead of spinal canal) contoured (not shown). Orange: GTV-pt. Magenta: CTV52.8 Gy. Pink: CTV59.4 Gy. Red: CTV66 Gy. Blue: PTV52.8 Gy-Exact*. Green: PTV2Exact*. Purple: PTV3Exact*. *PTVExacts denote PTVs collapsed inside the skin by 5 mm.

**Figure 4:**
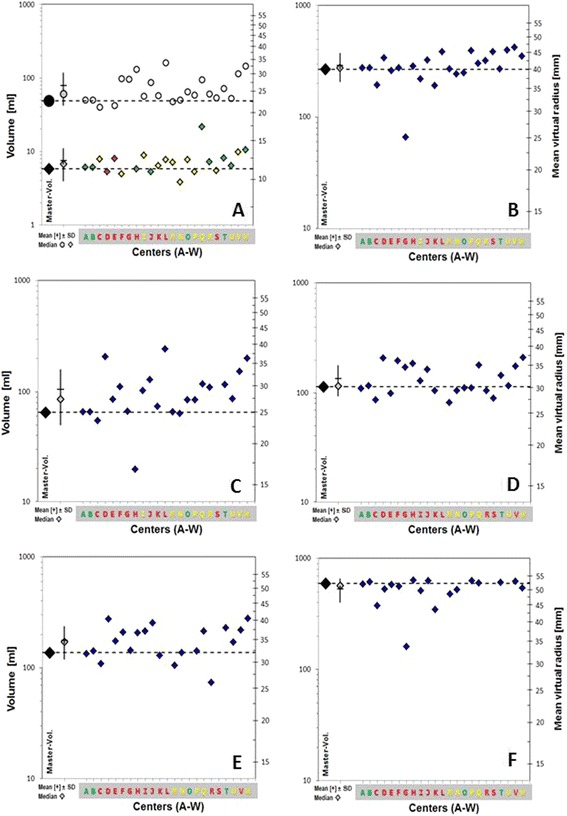
**Volumes (left y-axis) of delineated CTVs of the participating institutes (small symbols) in comparison to the master CTVs (large symbol and dashed lines).** The second symbols on the x-axis indicate mean volumes with standard deviation of the participating institutes (cross and solid lines) and median volumes of the participating institutes (gray symbols). The right y-axis indicates the mean virtual radius of the volumes. From the difference of the mean virtual radius of the institute’s volumes to the dashes lines one can estimate the average distance of the institute’s delineations to the master delineation. **(A)** CTV66 Gy (circle) (N = 23) and GTV-pt (diamond) (N = 22) Colour of GTV-pt symbols indicates grade (green = acceptable, yellow = borderline, red = unacceptable). **(B)** CTV52.8 Gy (N = 23). **(C)** CTV59.4 Gy (N = 22). **(D)** PTV66 Gy-Exact (N = 23). **(E)** PTV59.4 Gy-Exact (N = 20). **(F)** PTV52.8 Gy-Exact (N = 18). Colour of centres (A-W) indicates overall grade.

GTV-pt contours were evaluated as acceptable in 9/23 (39.1%) cases, borderline in 11 (47.8%) and unacceptable in three (13.0%). The specific LN with ECE was not reconstructed by 10/23 sites, but this was not by itself sufficient to downgrade the GTV if it was otherwise considered acceptable. Examples of submitted datasets with contours graded overall as minor or major deviation are shown in Figures 2 and 3 respectively. Volumetric indices of the CTVs are shown in Figures 4A-C as well as the online Additional file 1. Altogether, CTVs were termed acceptable in 4/23 (17.4%) datasets, borderline in 9 (39.1%) and unacceptable in 10 (43.5%). Contradicting protocol guidelines, 14/23 (60.9%) sites had at least one CTV crossing anatomic boundaries, 5/23 (21.7%) included all or part of level IA (ipsilateral or contralateral), 3/23 (13.0%) included the ipsilateral level V LN, 3/23 (13.0%) did not encompass all of the preoperative GTV, and 3/23 (13.0%) included the entire post-surgical bed. CTV contour grade was not predicted by GTV contour grade (p = 0.23). PTVs were considered acceptable in 4/23 cases (17.4%), borderline in 6 (26.1%) and unacceptable in 13 (56.5%) (Figures 2, 3, 4 D-F). Overall, 13.0% of sites’ target volumes (global evaluation of GTV, CTVs and PTVs) were acceptable, 43.5% graded as minor deviation and 43.5% as major deviation.

### Dosimetry

D98% and D95% constraints were used to evaluate whether sufficient RT dose was prescribed to the respective volumes (Online Additional file 1). Seventeen (73.9%) sites met both PTV52.8 Gy-Exact dose constraints. For the D98% constraint, there were six major deviations, and for the D95%, one minor and five major deviations. Twenty (87.0%) datasets met both PTV59.4 Gy-Exact constraints; three had major violations for both D98% and D95%. Based on the D98% of the PTV66 Gy-Exact, 21 were acceptable, with one minor and one major deviation. In terms of the D95%, 22 were acceptable, with one major violation. 21 (91.3%) DR datasets met both PTV66 Gy-Exact constraints. There was no statistical correlation between PTV contour evaluation and whether the site met PTV52.8 Gy-Exact, PTV59.4 Gy-Exact, or PTV66 Gy-Exact constraints (all p ≥ 0.74). Nine median PTV66 Gy-Exact doses exceeded 66 Gy by >2%. Whether the PTV66 Gy-Exact was evaluated as acceptable, borderline or unacceptable did not predict whether the median PTV66 Gy dose exceeded 66 Gy by 2% (p = 0.86). No plan exceeded a dose of 72.6 Gy to a volume larger than 1.8 cc outside the PTV66 Gy-Exact. In three datasets, optimization was probably performed on PTVs not collapsed inside the body contour. In one, a second non-IMRT plan used for treatment of the low neck was not submitted rendering the plan not fully evaluable.

### Organs at risk

**Figure 5:**
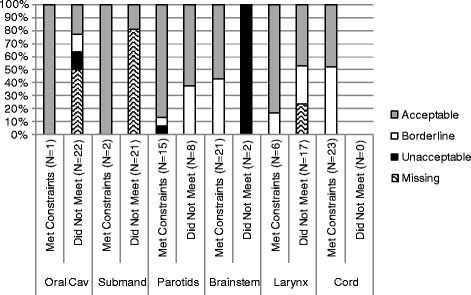
**Organ at risk contour evaluation and achievement of dose constraints.** Spinal cord, brainstem, and parotids were deemed mandatory for delineation and/or dose reporting and if one of these structures could not be assessed, it was called unacceptable. Remaining structures which were not submitted were called missing. Abbreviation: cord – spinal cord; submand – right submandibular gland; oral cav – oral cavity.

**Figure 6:**
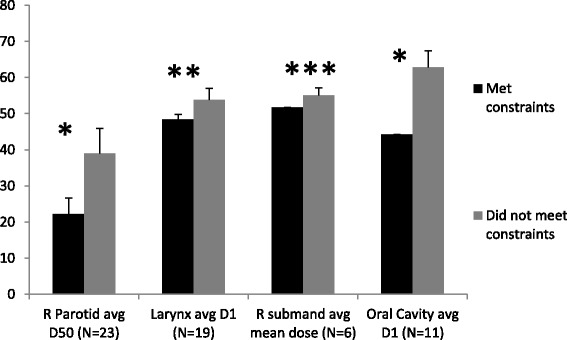
**Comparative average doses for organs at risk which met or did not meet dose constraints.** *p < 0.0001, **p < 0.01, ***p = 0.04. N denotes number of sites submitting a given structure. Abbreviations: avg – average; submand – submandibular gland.

Consensus contour evaluations are displayed subdivided by whether the OAR met dose constraints (Figure 5). If a mandatory OAR (spinal cord, brainstem, parotids) could not be assessed, it was rated as unacceptable. Other structures not submitted were rated as missing. Sites met dose constraints for an average of three OARs out of six (range 2–5). Recommended dose constraints were: a mean dose to either parotid <26 Gy (achieved by 7/23 sites); *or* ≥50% of either parotid receiving <30 Gy (achieved by 15/23); *or* a combined parotid D20cc of <20 Gy (achieved by 1/23). There was no statistically significant correlation between the volume of any CTV or PTV and ability to meet parotid dose constraints (all p > 0.20), or between parotid contour acceptability and meeting constraints (p = 0.17). Mean dose to the right parotid versus PTV volumes are shown in the online Additional file 1. The dose limit was met by one third of sites who contoured the (optional) right submandibular gland, although it was often included in the low dose PTV so was difficult to optimally spare. Recommended doses for both the oral cavity and larynx excluded overlap with PTV-Exacts. There were statistically significant correlations between contour grades and whether the site was able to meet respective dose constraints for the brainstem, right submandibular gland, oral cavity, and larynx (all p ≤0.004). Volumes of CTV58.4 Gy, CTV66 Gy and PTV58.4 Gy correlated significantly with left parotid D50 (all p ≤ 0.047), and volumes of CTV58.4 Gy and PTV58.4 Gy correlated significantly with left parotid mean dose (both p ≤ 0.043). Average doses were significantly higher for OARs which did not meet constraints versus those that did (p ≤ 0.04) (Figure 6).

## Discussion

Twenty of twenty-three GTVs, 13 CTVs and 10 PTVs were evaluated as acceptable or borderline, and three CTVs excluded part of the preoperative extent of tumour. Seventy-four percent, 87.0% and 91.3% of PTV52.8 Gy-, PTV59.4 Gy- and PTV66 Gy-Exacts, respectively, met all dose constraints. Although the overall degree of variation in target volume delineation was high, and centers’ volumes tended to be larger than the master contours (Figure 4) with the exception of elective nodal volumes, deviations were smaller in the high risk volumes (GTV-pt and CTV66 Gy). The mean distance of the GTV-pt contour from the master contour was, with the exception of one center, below 2.5 mm based on the mean virtual radius (Figure 4). Dosimetric data can be considered a proxy for the biologic effects of protocol treatment, had it been delivered [23], and since most locoregional recurrences occur in the high risk volume, this variation between centers would be expected to have a low impact on locoregional tumor control. The main causes of poor compliance with the guidelines were that the delineation of the nodal levels and the CTV margins on the GTV-pt were not performed entirely according to protocol.

Similar to the current study, investigators from the PARSPORT multicenter randomized trial were asked to delineate volumes as per protocol which were then compared centrally with a master set [24]. For three submissions, there were no significant differences between submitted and reference volumes; there were small discrepancies in four, attributed to the learning curve and inter-observer variability. Large differences from lack of adherence to the trial guidelines were found in the remaining three representing exclusion of specific anatomical areas or nodal levels. Planners also created PTVs based on CTV and OAR volumes provided [24]. Eight centres achieved the required dose constraints with the other two within 2%. Cord and brainstem tolerances were not exceeded in any plan. The average mean contralateral parotid dose was 25.5 Gy. All contralateral parotid doses <24 Gy were delivered with dynamic IMRT using 5 mm width leaves. The authors concluded that differences in parotid sparing were likely due to MLC leaf width rather than delivery technique; this information is not available for datasets in the present study [24].

A Swiss national DR-type study was undertaken to try to explain the reasons behind large discrepancies in target volume delineation [25]. Radiation oncologists from 11 centres received a CD containing a CT scan and MRI sequences for a 72 year old man with a well-differentiated T3N0 base of tongue HNSCC. The authors reported increased GTV homogeneity when more precise radiological imaging was available (contrast, thinner CT slices, multiple modalities). PTVs were more homogeneously defined, partially compensating for relative inconsistencies in GTV and CTV contours. The authors concluded that the main reasons for observed differences were variable interpretation of protocol instructions and ICRU definitions, particularly the CTV, and a compensation effect of the PTV where a clinical margin was subconsciously added [25]. In our study, median volumes of CTV52.8 Gy, CTV59.4 Gy and CTV66 Gy were on average 7%, 32%, and 24% larger than the respective master contours, suggesting that local investigators also tended to incorporate a safety margin.

Nelms et al. examined variation in OAR contours in a patient with oropharyngeal cancer to quantify inter-clinician variability as well as changes in IMRT dosimetry due strictly to OAR differences [26]. Investigators were provided with a CT dataset that included a pre-contoured GTV, three CTVs, and three PTVs. OAR definition was left to the discretion of local investigators; major variations were seen in resulting OAR sizes and shapes. The dosimetric impact of variation in OAR contours was estimated by overlying the reference OARs onto each site’s optimized dose grid. Reported dose differences depended on the degree of contour variation and the plan’s dose gradients with smaller differences seen with increasing OAR accuracy [26]. We provided multimodality imaging (preoperative CT and MRI) and references to OAR atlases to attempt to address this potential source of interobserver variation.

The ability to meet a dose-volume constraint is also dependent on the proximity of an OAR to a high-dose PTV or steep dose gradient [18,23,25-27]. The requirement to meet a single dose-volume constraint, considered a limitation in previous IMRT comparison studies [28], was avoided by provision of up to three constraints per structure in this trial. This alllows greater flexibility in how dose objectives can be met [18], although may increase plan heterogeneity.

A modern protocol should leave freedom in RT technique but clearly describe target volumes, dose homogeneity requirements, dose prescription, relative priorities, and dose-volume constraints to unambiguously specified OARs [29]. This study had a few limitations. A clearer distinction between requirements versus recommendations for OAR contours may have decreased the number of violations since absence of a mandatory structure resulted in an ‘unacceptable’ rating. Although both CT and MRI were provided for the DR patient, 18 F-FDG PET was not utilized. Preoperative PET images may have helped delineation of the GTV and therefore increased inter-observer agreement [30,31]. Regular progress meetings for clinicians using the same TPS and hardware for structured knowledge transfer, as suggested by Clark et al. [24] were not performed. In evaluating acceptability of target volumes, a reference must be defined. It could be argued whether our set of master contours was worthy of being the gold standard, but as per Nelms et al., given any reference dataset, variability would still exist [26]. Finally, inter-centre comparison of OAR doses was complicated by the fact that each centre created its own contours, making it harder to determine the degree to which differences were influenced by planning technique.

Steps have already been taken to address the last issue in the form of a two-step DR procedure. First, the DR case is sent to participating centres for delineation of volumes, along with a list of requirements for compliance and predefined major deviations. Via an iterative process, local investigators resubmit volumes for evaluation until no major deviations are identified. Minor deviations are also reported to participating centres but correction is not required. In the second step, the master contours are sent to centres, based on which a protocol-compliant treatment plan is generated and centrally reviewed. This guarantees the same starting conditions for all centres. Additional means by which improvement in DR quality could be achieved would depend on determining specific reasons for non-compliance within each trial; potential reasons have been recently reviewed [19].

## Conclusions

Wide variation in dose planning in the EORTC 22071 dummy run confirms the complexity of development of IMRT-based multicentre clinical studies for head and neck cancer, and underscores the need for insistence on adherence to strict QA procedures for the present time.

## Additional file

Additional file 1:
**Dummy run case. Table S1.** Volume and dose statistics for CTVs and PTVs. Dosimetric indices for CTVs not prespecified in protocol. Abbreviations: avg – average; N/a – not applicable; SD – standard deviation. **Figure S1.** Mean dose to right parotid versus planning target volumes. **(A)** PTV52.8 Gy **(B)** PTV59.4 Gy **(C)** PTV66 Gy.

## Abbreviations

CRT: Chemoradiotherapy
CT: Computed tomography
CTV: Clinical target volume
DICOM: Digital imaging and communications in medicine
DFS: Disease-free survival
DR: Dummy run
DVH: Dose-volume histogram
ECE: Extra-capsular extension
EGFR: Epidermal growth factor receptor
EORTC: European Organization for Research and Treatment of Cancer
GTV: Gross tumour volume
GTV-pt: Preoperative extent of primary tumour
Gy: Gray
HNSCC: head and neck squamous cell carcinoma
ICRU: International Commission on Radiation Units and Measurements
IMRT: Intensity-modulated radiotherapy
LN: Lymph node
MRI: Magnetic resonance imaging
OAR: Organ at risk
OS: Overall survival
PTV: Planning target volume
PTV-Exact: Optimization structure
QA: Quality assurance
ROG: Radiation oncology group
RT: Radiotherapy
RTOG: Radiation therapy oncology group
SIB: Simultaneous integrated boost
TPS: Treatment planning system

## References

[1] Huang DT, Johnson CR, Schmidt-Ullrich R, Grimes M (1992). Postoperative radiotherapy in head and neck carcinoma with extracapsular lymph node extension and/or positive resection margins: a comparative study. Int J Radiat Oncol Biol Phys.

[2] Kokal WA, Neifeld JP, Eisert D, Lipsett JA, Lawrence W, Beatty JD, Parker GA, Pezner RD, Riihimaki DU, Terz JJ (1988). Postoperative radiation as adjuvant treatment for carcinoma of the oral cavity, larynx, and pharynx: preliminary report of a prospective randomized trial. J Surg Oncol.

[3] Marcial VA, Pajak TF, Kramer S, Davis LW, Stetz J, Laramore GE, Jacobs JR, Al-Sarraf M, Brady LW (1988). Radiation therapy oncology group studies in head and neck cancer. Semin Oncol.

[4] Sadeghi A, McLaren J, Grist WL, Tran L, Kuisk H (1986). Value of radiation therapy in addition to surgery for cancer of the head and neck. Otolaryngol Head Neck Surg.

[5] Fietkau R, Lautenschläger C, Sauer R, Dunst J, Becker A, Baumann M, Wendt K, Gruschow K, Hess C, Budach V, Iro H (2006). Postoperative concurrent radiochemotherapy versus radiotherapy in high-risk SCCA of the head and neck: results of the german phase III trial ARO 96–3 [abstract]. J Clin Oncol.

[6] Bernier J, Domenge C, Ozsahin M, Matuszewska K, Lefebvre JL, Greiner RH, Giralt J, Maingon P, Rolland F, Bolla M, Cognetti F, Bourhis J, Kirkpatrick A, van Glabbeke M, European Organization for Research and Treatment of Cancer Trial 22931 (2004). European organization for research and treatment of cancer trial 22931. Postoperative irradiation with or without concomitant chemotherapy for locally advanced head and neck cancer. N Engl J Med.

[7] Cooper JS, Pajak TF, Forastiere A, Ozsahin EM, Jacobs JR, Jassem J, Ang KK, Lefebvre JL (2004). Radiation therapy oncology group 9501/intergroup. Postoperative concurrent radiotherapy and chemotherapy for high-risk squamous-cell carcinoma of the head and neck. N Engl J Med.

[8] Peters LJ, Goepfert H, Ang KK, Byers RM, Maor MH, Guillamondegui O, Morrison WH, Weber RS, Garden AS, Frankenthaler RA (1993). Evaluation of the dose for postoperative radiation therapy of head and neck cancer: first report of a prospective randomized trial. Int J Radiat Oncol Biol Phys.

[9] Bernier J, Cooper JS, Pajak TF, van Glabbeke M, Bourhis J, Forastiere A, Ozsahin EM, Jacobs JR, Jassem J, Ang KK, Lefebvre JL (2005). Defining risk levels in locally advanced head and neck cancers: a comparative analysis of concurrent postoperative radiation plus chemotherapy trials of the EORTC (#22931) and RTOG (# 9501). Head Neck.

[10] Bonner JA, Harari PM, Giralt J, Azarnia N, Shin DM, Cohen RB, Jones CU, Sur R, Raben D, Jassem J, Ove R, Kies MS, Baselga J, Youssoufian H, Amellal N, Rowinsky EK, Ang KK (2006). Radiotherapy plus cetuximab for squamous-cell carcinoma of the head and neck. N Engl J Med.

[11] Fairchild A, Straube W, Laurie F, Followill D (2013). Does quality of radiotherapy predict outcomes of multicentre cooperative group trials? A literature review. Int J Radiat Oncol Biol Phys.

[12] Ohri N, Shen X, Dicker A, Doyle L, Harrison A, Showalter T (2013). Radiotherapy protocol deviations and clinical outcomes: a meta-analysis of cooperative group clinical trials. J Natl Cancer Inst.

[13] Weber D, Tomsej M, Melidis C, Hurkmans C (2012). QA makes a clinical trial stronger: evidence-based medicine in radiation therapy. Radiother Oncol.

[14] Abrams RA, Winter KA, Regine WF, Safran H, Hoffman JP, Lustig R, Konski AA, Benson AB, Macdonald JS, Rich TA, Willett CG (2012). Failure to adhere to protocol specified radiation therapy guidelines was associated with decreased survival in RTOG 9704–a phase III trial of adjuvant chemotherapy and chemoradiotherapy for patients with resected adenocarcinoma of the pancreas. Int J Radiat Oncol Biol Phys.

[15] Peters LJ, O’Sullivan B, Giralt J, Fitzgerald TJ, Trotti A, Bernier J, Bourhis J, Yuen K, Fisher R, Rischin D (2012). Critical impact of radiotherapy protocol compliance and quality in the treatment of advanced head and neck cancer: results from TROG 02.02. J Clin Oncol.

[16] Weber D, Poortmans P, Hurkmans C, Aird E, Gulyban A, Fairchild A (2011). Quality assurance for prospective EORTC radiation oncology trials: the challenges of advanced technology in a multicentre international setting. Radiother Oncol.

[17] Ibbott G, Followill D, Molineu H, Lowenstein J, Alvarez P, Roll J (2008). Challenges in credentialing institutions and participants in advanced technology multi-institutional clinical trials. Int J Radiat Oncol Biol Phys.

[18] Williams M, Bailey M, Forstner D, Metcalfe P (2007). Multicentre quality assurance of intensity-modulated radiation therapy plans: a precursor to clinical trials. Australas Radiol.

[19] Fairchild A, Collette L, Hurkmans C, Baumert B, Weber DC, Gulyban A, Poortmans P (2012). Do results of the EORTC dummy run predict quality of radiotherapy delivered within multicentre clinical trials?. Eur J Cancer.

[20] Ang K, Zhang Q, Rosenthal D, Nguyen-Tan PF, Sherman EJ, Weber RS, Galvin JM, Bonner JA, Harris J, El-Naggar AK, Gillison ML, Jordan RC, Konski AA, Thorstad WL, Trotti A, Beitler JJ, Garden AS, Spanos WJ, Yom SS, Axelrod RS (2011). A randomized phase III trial (RTOG 0522) of concurrent accelerated radiation plus cisplatin with or without cetuximab for stage III-IV head and neck squamous cell carcinomas [Abstract]. J Clin Oncol.

[21] Gregoire V, Eisbruch A, Hamoir M, Levendag P (2006). Proposal for the delineation of the nodal CTV in the node-positive and post-operative neck. Radiother Oncol.

[22] ICRU Report 83 (2010). Prescribing, recording and reporting photon beam intensity-modulated radiotherapy. J ICRU.

[23] Das I, Cheng C, Chopra K, Mitra R, Srivastava S, Glatstein E (2008). Intensity-modulated radiation therapy dose prescription, recording and delivery: Patterns of variability among institutions and treatment planning systems. J Natl Cancer Inst.

[24] Clark C, Miles A, Guerrero Urbano M, Bhide SA, Bidmead AM, Harrington KJ, Nutting CM (2009). Pre-trial quality assurance processes for an intensity-modulated radiation therapy trial: PARSPORT, a UK multicentre phase III trial comparing conventional radiotherapy and parotid-sparing IMRT for locally advanced head and neck cancer. Brit J Radiol.

[25] Moeckli R, Sozzi W, Mirimanoff R, Ozsahin M, Zouhair A, Valley JF, Bochud F (2009). Physical considerations on discrepancies in target volume delineation. Z Med Phys.

[26] Nelms B, Tome W, Robinson G, Wheeler J (2012). Variations in the contouring of organs at risk: test case from a patient with oropharyngeal cancer. Int J Radiat Oncol Biol Phys.

[27] Chao K, Low D, Perez C, Purdy J (2000). Intensity-modulated radiation therapy in head and neck cancers: the mallinkrodt experience. Int J Cancer.

[28] Skala M, Holloway L, Bailey M, Kneebone A (2005). Australia-wide comparison of intensity modulated radiation therapy prostate plans. Australas Radiol.

[29] Fairchild A, Bar-Deroma R, Collette L, Haustermans K, Hurkmans C, Lacombe D, Maingon P, Poortmans P, Tomsej M, Weber DC, Gregoire V (2012). Development of clinical trial protocols involving advanced radiation therapy techniques: the EORTC radiation oncology group approach. Eur J Cancer.

[30] Troost E, Schinagl D, Bussink J, Oyen W, Kaanders J (2010). Clinical evidence on PET-CT for radiation therapy planning in head and neck tumours. Radiother Oncol.

[31] Koshy M, Paulino A, Howell R, Schuster D, Halkar R, Davis L (2005). F-18 FDG PET-CT fusion in radiotherapy treatment planning for head and neck cancer. Head Neck.

