# Supplementary material for: Quality assurance for the EORTC 22071–26071 study: dummy run prospective analysis
Source: Radiat Oncol. 2014 Nov 26;9:248. doi: 10.1186/s13014-014-0248-9 (PMC4311463; doi:10.1186/s13014-014-0248-9)
Supplement: Additional file 1: — Dummy run case. Table S1. Volume and dose statistics for CTVs and PTVs. Dosimetric indices for CTVs not prespecified in protocol. Abbreviations: avg – average; N/a – not applicable; SD – standard deviation. Figure S1. Mean dose to right parotid versus planning target volumes. (A) PTV52.8 Gy (B) PTV59.4 Gy (C) PTV66 Gy. [file 13014_2014_248_MOESM1_ESM.docx]

**Proposed Web Supplements**

**DR Case**

The DR patient was 51 years old with Karnofsky performance status of 90 and a palpable 1cm left level IIA LN. Panendoscopy revealed a 20x15 mm ulcerative lesion on the left lateral tongue, confirmed on biopsy as moderately differentiated squamous cell carcinoma. Contrast-enhanced diagnostic CT scan of the neck, chest and abdomen demonstrated only the tongue lesion with two suspicious left level II nodes. MRI neck measured the primary lesion at 26x15 mm and the lymph nodes at 10 mm and 11 mm. The patient underwent en bloc resection, partial glossectomy and left modified radical neck dissection with reconstruction (free radial forearm flap). Pathology report confirmed a moderately differentiated 27 mm squamous cell carcinoma of the left lateral tongue with a depth of invasion of 18 mm. Medial and lateral margins were positive. Of 29 lymph nodes removed, one level IIA lymph node was involved, measuring 23mm in size with ECE. There was no angio- or perineural invasion.

**Additional file 1: Table S1**

Volume and dose statistics for CTVs and PTVs. Dosimetric indices for CTVs not prespecified in protocol. Abbreviations: avg – average; N/a – not applicable; SD – standard deviation.

|  | **CTV52.8Gy** | **CTV59.4Gy** | **CTV66Gy** | **PTV52.8Gy** | **PTV59.4Gy** | **PTV66Gy** |
| --- | --- | --- | --- | --- | --- | --- |
| **Average Volume** | **N=23** | **N=22** | **N=23** | **N=18** | **N=20** | **N=22** |
| Reference volume  Mean volume  SD  Minimum volume  Maximum volume | 268.5cc  290.7cc  81.4cc  66.0cc  423.0cc | 64.5cc  104.2cc  54.4cc  19.6cc  242.1cc | 49.0cc  79.5cc  37.1cc  39.9cc  161.6cc | 595.5cc  529.0cc  124.3cc  160.0cc  632.2cc | 136.9cc  177.9cc  58.6cc  73.5cc  279.9cc | 114.3cc  136.8cc  42.1cc  82.0cc  211.1cc |
| **Average Dose** |  |  |  |  |  |  |
| Minimum (D99%)  D98%  D95%  Median (D50%)  Maximum (D1%) | N/a | N/a | N/a | 48.6Gy  50.3Gy  51.9Gy  56.9Gy  68.6Gy | 57.9Gy  59.0Gy  60.6Gy  65.9Gy  69.6Gy | 62.2Gy  63.2Gy  64.3Gy  67.0Gy  70.1Gy |

**Additional file 1: Figure S1**

Mean dose to right parotid versus planning target volumes. (A) PTV52.8Gy (B) PTV59.4Gy (C) PTV66Gy.

A.

B.

C.
